# The Effects of Intravitreal Administration of Antifungal Drugs on the Structure and Mechanical Properties Peripheral Blood Erythrocyte Surface in Rabbits

**DOI:** 10.3390/ijms231810464

**Published:** 2022-09-09

**Authors:** Maria N. Starodubtseva, Sofia Karachrysafi, Nastassia M. Shkliarava, Irina A. Chelnokova, Dimitrios Kavvadas, Kyriaki Papadopoulou, Paraskevi Samara, Vasileios Papaliagkas, Antonia Sioga, Anastasia Komnenou, Vasileios Karampatakis, Theodora Papamitsou

**Affiliations:** 1 Institute of Radiobiology of the NAS of Belarus, 4, Fedyuninskogo St., 246007 Gomel, Belarus; 2 Medical Department, Gomel State University, 5, Lange St., 246000 Gomel, Belarus; 3 Laboratory of Histology, Embryology, Medical Department, Faculty of Health Sciences, Aristotle University of Thessaloniki, 54124 Thessaloniki, Greece; 4 MRes Applied Health Research, Department of Health Sciences, University of Leicester, Leicester LE1 7RH, UK; 5 Department of Biomedical Sciences, School of Health Sciences, International Hellenic University, 57400 Thessaloniki, Greece; 6 School of Veterinary Medicine, Faculty of Health Sciences, Aristotle University of Thessaloniki, 57400 Thessaloniki, Greece; 7 Laboratory of Experimental Ophthalmology, Medical Department, Faculty of Health Sciences, Aristotle University of Thessaloniki, 57400 Thessaloniki, Greece

**Keywords:** antifungal agents, micafungin, voriconazole, erythrocytes, atomic force microscopy

## Abstract

Background: Fungal infections can pose great threat to sight. Immediate treatment is usually required; antifungal agents are widely accepted and are effective in most cases. The present experimental study aims to investigate the probable effects of intravitreal injection of antifungal agents on the structure and mechanical properties of the surface of peripheral blood erythrocytes. Methods: Nine albino New Zealand white rabbits, aged five months old, were chosen for the experiment. Solutions of micafungin, voriconazole, or balanced salt solution (BSS) were injected into the midvitreous. Animals were divided into two experimental groups and one control group. Blood sampling from an intravenous (IV) line was performed after 10 days from the last IV injection. An atomic force microscope (AFM) was used to study the structural and mechanical properties of cell surfaces. Results: The analysis results showed that the parameters of the cytoskeleton’s spatial organization changed insignificantly with the antifungal drug treatment. Conclusions: Our findings suggest that locally administered antifungal drugs can cause significant changes to the structure and frictional properties of the erythrocyte surface. These effects occur in the long-term period after administration of the drugs and represent a potential possibility for violation of blood supply to tissues, and the further development of negative side effects.

## 1. Introduction

The frequency of fungal infections is increasing every year, which is a significant medical and social problem worldwide [1]. Fungal endophthalmitis is a serious vision-threatening infection [2]. Intravitreal administration of antifungal agents is the main therapeutic approach [3]. For the treatment of mycoses, drugs that differ by origin, mechanism of action, indications for use, and methods of administration have been used, often leading to highly toxic side effects [4].

Side effects of micafungin treatment are known to include microangiopathic hemolytic anemia and thrombocytopenia with microvascular thrombosis [5]. One possible mechanism of thrombosis development involves eryptosis, i.e., the suicidal death of erythrocytes characterized by cell shrinkage and cell membrane scrambling with phosphatidylserine translocation to the erythrocyte surface [5]. Peter et al. showed that micafungin triggers hemolysis and eryptosis with cell shrinkage and phospholipid scrambling of the erythrocyte cell membrane [5]. There is no evidence in the literature presenting similar behavior due to voriconazole intake. Generally, voriconazole is associated with increased risk of cutaneous malignancies, phototoxicity, hallucinations, peripheral neuropathy, alopecia, hyponatremia and in some cases, toxicity [6]. Regarding the adverse ophthalmic outcomes of present antifungal agents, ultrastructural evaluation in the retina of animals after intravitreal administration of voriconazole and micafungin revealed several lesions in retinal layers [3]. These alterations were not visible with optical microscopy, but the detection of IL-8 and the microscopic morphological lesions implicate possible adverse outcomes on retina, even in the safe dosage range of the two antifungal agents [3].

Antifungal drugs introduced locally can act on blood cells, changing their properties, potentially contributing to the drugs’ negative side effects [5].

Atomic force microscopy (AFMhas been used to study various properties of the cell surface at nano- and microscale levels, including the parameters of cell surface morphology, elastic, friction, and adhesive properties [7,8,9,10,11,12]. It can operate either in air for chemically fixed cells or in liquids for living cells, over a wide range of temperatures, and requires relatively simple procedures to prepare cell samples without labeling with fluorescentor other dyes. The frictional properties (mechanical material properties) of the cell surface, which are determined by the molecular and atomic structure of the surface, are important not only for the functioning of cells in an organism, but are also informative markers of the composition and state of the cell surface [7,9]. AFM high-resolution three-dimensional images of cell surfaces can be obtained by monitoring the interaction forces between an AFM probe and the surface. In contact mode, in which the AFM probe is kept in contact with the surface while scanning, is possible simultaneously record several AFM images, including topographical images and property maps, among them a friction force map.

The purpose of the study was to reveal the changes in the structure and mechanical properties of the surface of peripheral blood erythrocytes at nano- and microscale levels after intravitreal (IV) administration of two antifungal agents, Micafungin and Voriconazole, in an animal model.

## 2. Results

Administration of antifungal drugs to rabbits led to a significant change in the distribution of erythrocyte shapes in the blood; for double administration of micafungin (ΜM) spherocytosis values were up to 19 ± 6% (95% CI), and for sequential administration of micafungin and voriconazole (VM) echinocytosis values up to 55 ± 25% (95% CI) were revealed. 

**Figure 1:**
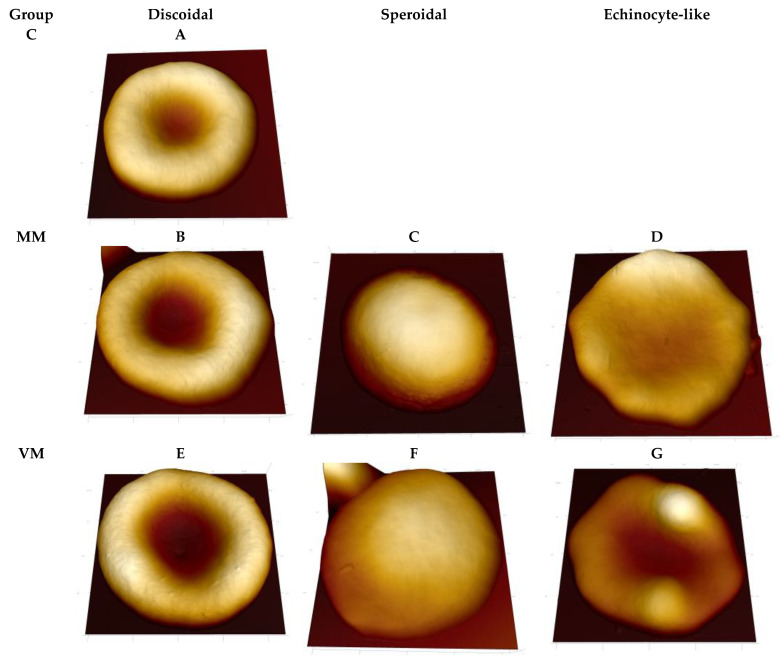
3D topographical images of rabbit erythrocytes from different experimental groups. C, control group; ΜM, two intravitreal injections of micafungin; VM, one intravitreal injection of voriconazole (on day 0) and one intravitreal injection of micafungin (on day 4). Scan sizes are (**A**) 6.5 μm × 6.5 μm; (**B**) 6.4 μm × 6.4 μm; (**C**) 6.0 μm × 6.0 μm; (**D**) 7.5 μm × 7.5 μm; (**E**) 6.5 μm × 6.5 μm; (**F**) 6.3 μm × 6.3 μm; (**G**) 6.4 μm × 6.4 μm.

Figure 1 represents three-dimensional AFM topographical images of rabbit erythrocytes from different experimental groups.

For further analysis of the nanoscale features of the cell surface and their changes after antifungal drug administration, we used erythrocytes of discoidal shapes from each group (Figure 1A,B,E). Overall morphological features of the discoidal erythrocytes of different groups were similar at the microscale. This fact allowed us to standardize cases and focus on nanoscale cell-surface defects. The groups consisted of controls, two with intravitreal injections of micafungin (ΜM), and one with intravitreal injection of voriconazole (VM). According to AFM data, the averaged diameters of erythrocytes with discoidal form in Control (C), ΜM, and VM groups were 6.01 ± 0.28 μm, 6.18 ± 0.17 μm, and 6.18 ± 0.21 μm, respectively (95% CI, *n* = 20–30).

**Figure 2:**
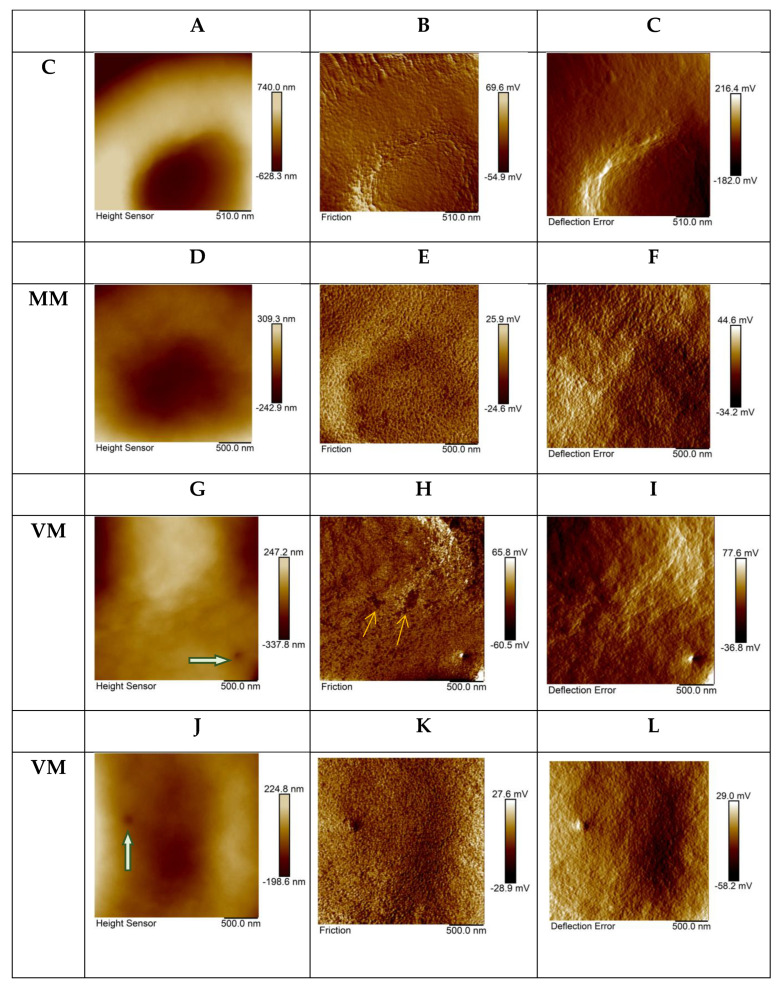
The topography and property maps (friction and deflection error) for microscale areas of erythrocyte surface from different experimental groups. C, control group; ΜM, two intravitreal injections of micafungin; VM, one intravitreal injection of voriconazole (on day 0) and one intravitreal injection of micafungin (on day four). Topographic maps (**A**,**D**,**G**,**J**), friction force maps (**B**,**E**,**H**,**K**), deflection error maps (**C**,**F**,**I**,**L**). Scan size 2.5 μm × 2.5 μm, resolution 256 × 256 pixels.

When analyzing AFM images of small areas of the erythrocyte surface in animals after antifungal drug administration, qualitative and quantitative changes were detected in the parameters of the distribution of sliding friction forces between the tip of the AFM probe and the cell surface. On the surface of many cells, the presence was observed of membrane defects such as holes or small areas with frictional properties sharply distinguished from those of their surroundings. Sizes of defects were about 30–200 nm. Figure 2G–L represents in microscale areas of the VM group’s erythrocyte surface with defects in the form of single holes, indicated with thick green arrows on topographic images of the erythrocyte surface (Figure 2G,J) and “spots” (small surface areas) with differences in friction properties, indicated with thin yellow arrows on the friction-force maps (Figure 2H,K).

Quantitative comparison of AFM image parameters for microscale areas of the cell surface in the control sample and the studied samples showed significant differences in roughness in the topographic images and property maps. The roughness of the topographic maps was reduced in the ΜM and VM groups (Table 1). Microscale areas of the cell surface became smoother after treatment with the drug. At the same time, the spatial distribution of friction forces for the same sections of the cell surface became more heterogeneous. Meanwhile, the roughness of friction-force maps of both study samples was significantly higher than the corresponding parameter of the control sample, not only for the discocytes, but also for the echinocytes.

The cell-surface property maps at the microscale represent the periodic surface showing 2D spatial network related to erythrocyte actin–spectrin network (Figure 2, friction and deflection-error maps). The spatial period of these maps corresponded to a cytoskeleton network cell (unit) [7]. We applied PSD analysis to the property maps [7] to reveal the average size of the cytoskeleton network (Table 2). Complex periodograms were fitted as a function that represented a sum of three Gauss functions. Gaussian peaks obtained for periodograms were associated with the main types (subpopulations) of periodical structures on the maps of the erythrocyte surface layer’s mechanical properties [7]. According spatial spectral analysis results, the discoidal shape of erythrocytes was characterized by the presence of three populations of cytoskeleton units (cells) of various sizes; about 43–46 nm, about 82–94 nm, and about 161–206 nm. These results corresponded to those obtained for human erythrocytes with hereditary spherocytosis [8]. In our study, it was found that for discoidal erythrocytes the percentage of membrane skeleton units of a small size was higher than in the case of human discocytes in hereditary spherocytosis. This may be due to specific features of either the organism type or the organism’s pathology. In terms of the topic discussed in this paper, it is interesting that the results of spatial spectral analysis showed that the parameters of the spatial organization of the cytoskeleton changed slightly after administration of antifungal drugs. This shows that it is unlikely that antifungal drugs had a strong effect on cytoskeletal proteins, and it is more probable that the main effect of the drugs was directed to the cell membrane.

## 3. Discussion

Most of the data on the effectiveness and side effects of antifungal drugs have been obtained in animal studies. There are only limited data from human tissue biopsies, including samples taken during surgery or autopsy [13]. In the present work a rabbit animal model was used for revealing the effects of local administration of two antifungal drugs on the state and properties of peripheral blood erythrocytes. This research is important for understanding the mechanisms and genesis of the drugs’ side effects.

The mechanism of action of voriconazole is similar to that of all azole agents; the inhibition of cytochrome P450 (CYP 450)-dependent 14α-lanosterol demethylation, which is a vital step in cell membrane ergosterol synthesis by fungi [3,14]. In contrast to voriconazole, micafungin is a weak inhibitor of the enzymes of the cytochrome P 450 (CYP) family [3,13]. Micafungin is a strong inhibitor of multidrug resistance protein 4 and a mild inhibitor of multidrug resistance proteins 1 and 5, and breast cancer resistance protein [3,14]. The mechanism of CYP 450 inhibition by azole antifungals includes their binding to the haem enzyme group. Voriconazole can be metabolized by cytochrome P450 isotypes CYP2C19, CYP3A4, and CYP2C9 through common metabolic pathways for many drugs, for example, proton pump inhibitors [15,16,17]. Omeprazole is a proton pump inhibitor; prolonged use of omeprazole may be associated with anemia. The eryptotic, oxidative, and hemolytic effects of therapeutical doses (0.5, 1 and 1.5 μm) of omeprazole were revealed after exposing erythrocytes in vitro for 48 h [14]. Omeprazole was found to induce reduction in superoxide dismutase, glutathione peroxidase, and catalase activities, as well as causing hemolysis and triggering erythrocytes’ phosphatidylserine exposure and certain other features of eryptosis [14].

Therefore, both antifungal drugs used in our study are potential stimulators of eryptosis, contributing to changes in cell volume, shape, and composition of the cellular membrane. Our study revealed the poikilocytosis of erythrocytes, with prevailing shapes as echinocytes and spheroidal cells without increase in cell sizes. These findings are evidence of the possibility of eryptosis in peripheral blood, even in cases of local drug administration.

Detailed analysis of nano-architectonics and nanomechanical properties of erythrocyte surfaces showed significant changes of those properties at the nanoscale level, even for discoidal erythrocytes. The antifungal drug effect on the cytoskeleton (actin–spectrin network) seems to be minor. The parameters of the spatial structure of the actin–spectrin network changed slightly after drug treatment. In the eryptosis process, phosphatidylserine is translocated from the inner leaflet to the external leaflet of the plasmalemma. Phosphatidylserine is one of the most prevalent naturally occurring negatively charged membrane phospholipids. Changing the lipid composition of the external leaflet in the cellular membrane can change the spatial distribution of surface charge and the mechanical forces that can influence the elastic, adhesion, and friction properties of the erythrocyte surface. The introduction of phosphatidylserine into one leaflet of the lipid bilayer was shown in numerical simulation experiments to reduce the bilayer’s bending rigidity [17]. Nguyen et al. [18] carried out molecular dynamics simulation experiments and demonstrated that overexpression of the phosphatidylserine lipids in the outer leaflet could not significantly alter the area per lipid, the membrane thickness, the lipid order parameters, or the elasticity moduli of the cancer membranes. However, a reduction in the cholesterol concentration could lead to clear changes in those quantities, especially decreases in the bending, tilt, and twist moduli [18].

### Limitations and Future Studies

There are limitations in the present research that should be addressed in future experimental studies on the matter. The fact that there was no peripheral blood evaluation was our main limitation. Because only small doses of drugs were administrated locally, the drug plasma concentration in the animals should be significantly lower than that observed in patients following treatment. For instance, 25 μg micafungin was administrated into the rabbit’s eye. Since there is more than 120 mL blood in one experimental rabbit (60 mL/kg), the drug plasma concentration should be less than 0.3 μg/mL, which is equivalent to 70 time less than the drug concentration observed in plasma from patients following treatment [19]. However, our findings suggest that regardless of the modification of the drug’s concentration in the peripheral blood, applied through intravitreal administration, the experimental specimens presented differences in comparison to the control, strongly suggestive of changes having been induced in the erythrocytes’ shapes. A comparative study between intravitreal and direct systematic administration of the drug should be performed in future to further clarify the concentrations responsible for the adverse outcomes in the erythrocytes. Also, future studies should consider the sex of the experimental animals.

## 4. Materials and Methods

### 4.1. Animals and Antifungal Drugs Administration Conditions

All animal procedures were approved by the Department of Animals’ Health and Veterinary Drug Use Committee (protocol number 57253(211)) and conducted in accordance with the ARVO statement on the Use of Animals in Ophthalmic and Vision Research.

Nine albino New Zealand White Rabbits (2–4 kg body weight), aged five months old, were chosen for the experiments. Male and female rabbits were used. The rabbits were housed in specially designed cages, made of carefully selected materials that did not affect the animals’ health, with easy access for everyday cleanliness and care. This included unlimited access to fresh, clean water, condensed aliment, and hay, which were daily renewed. The cages were equipped with a ventilation system in order to maintain stable temperature, and with a lightning regulation system.

Solutions of micafungin, voriconazole, or balanced salt solution (BSS) were injected into the midvitreous section. Considering that a rabbit’s average vitreous volume is approximately 1.6 mL [20,21,22,23,24], voriconazole was administered at a dose 40 μg/0.1 mL to achieve an intravitreal concentration of 25 μg/mL. Micafungin was administered at a dose of 25 μg/0.1 mL.

Animals were divided to two experimental groups and one control group. Micafungin or voriconazole were intravitreally administered into the right eye of each rabbit in the experimental groups. In the left eye of each rabbit, BSS was intravitreally administered. Each group consisted of three animals. In Group ΜM, two intravitreal injections of micafungin were administered into the right eye (the first on day 0 and the second on day four) and two intravitreal injections of BSS were administered into the left eye of each rabbit (the first on day 0 and the second on day four). In Group VM, one intravitreal injection of voriconazole (on day 0) and one intravitreal injection of micafungin (on day four) were administered into the right eye and two intravitreal injections of BSS (the first on day 0 and the second on day 4) were administered into the left eye of each rabbit. Euthanasia of the animals in Group ΜM and VM was performed on day 14. In the control group, two intravitreal injections of BSS (the first on day 0 and the second on day four) were administered into both eyes of each rabbit.

The rabbits were weighed and anesthetized using dexmedetomidine 50 μg/kg and butorphanol 0.1 mg/kg (i.m.) for pre-narcosis, as well as ketamine 25 mg/kg i.m. and propofol 0.5 mg/kg i.v. (wherever more time for successful anesthesia was required). The surface of the eye was anesthetized with proparacaine hydrochloride 0.5%. The mydriasis of the pupils was induced by tropicamide 1%. The voriconazole dosage 40 μg/0.1 mL, micafungin dosage 25 μg/0.1 mL, and BSS were administered to the center of the vitreal body via a 30 gauge needle. After the injection, a cotton patch was placed over the site for 30 s to avoid any leakage. Ophthalmological examination under a slit lamp and indirect ophthalmoscopy were performed to all rabbits before the euthanasia. In addition, blood samples were taken from all rabbits before euthanasia. The euthanasia was performed with the use of dexmedetomidine, ketamine, propofol, and KCl.

### 4.2. Preparation of Erythrocyte Samples for AFM

Blood sampling from an intravenous (IV) line was performed after 10 days from the last IV injection. Thin glass slides (8 μm × 8 μm × 1 μm) were cleaned and stored prior to sample cell preparation in 70% ethanol. Erythrocytes were isolated from blood and fixed with 1% glutaraldehyde (37 °C, 15 min) in a suspension. The drops of the diluted erythrocyte suspension were placed on the glass slides, forming a monocellular layer when they spread, and were dried at room temperature.

### 4.3. Atomic Force Microscopy

A Bruker BioScope Resolve atomic force microscope (AFM) was used to study the structural and mechanical properties of the cell surfaces. The scanning procedure was performed according to the workflow proposed by the AFM manufacturer. Scanning in air was performed using a DNP-10 probe (Bruker, https://www.brukerafmprobes.com (accessed on 1 June 2022)), level D, with spring constant of 0.06 N/m and tip radius of 20 nm in contact mode at room temperature. The signal during the scanning process was simultaneously recorded in three channels, with the formation of three maps of surface properties: High, deflection error, and friction. The first two maps provided information about the structural features of the studied surface, the latter provided additional information about the mechanical properties (frictional properties) of the surface. The surfaces of the whole cells were scanned at a scan size of 10 μm × 10 μm, scanning rate 0.5 Hz, and resolution 256 × 256 pixels. The small areas of the cell surface were scanned over three different cell zones each, using ten cells per sample. For this, the scan size was 2.5 μm × 2.5 μm, scanning rate 0.3 Hz, and resolution 256 × 256 pixels.

### 4.4. AFM Data Analysis

Images were simultaneously recorded in contact mode and were included in the further statistical analysis, including topographic images (topography, height), maps of friction forces (friction), and deflection error (deflection error). For the property maps of the erythrocyte surface, the roughness was measured and power spectral density (PSD) analysis was carried out using NanoScope Analysis software. The Flatten command was used prior to image analysis commands.

The statistical samples of roughness for the C, ΜM, and VM groups were formed by measuring three arbitrary small areas of 1 μm × 1 μm size selected from each recorded area of 2.5 μm × 2.5 μm, for about seven different discoidal erythrocytes from each group. The data were checked using the Kolmogorov–Smirnov test for compliance with the normal distribution law. The data were represented as the median and limits of the interquartile range (Me(LQ; UQ)). The Mann–Whitney U test was employed to compare the sample parameters. The PSD analysis provided a representation of the amplitude of a surface’s roughness as a function of the spatial frequency of the roughness; spatial frequency is the inverse of the wavelength of the roughness features (spatial period, T). The PSD curves for the deflection-error maps were approximated by three Gaussian functions using OriginPro 8.0 software.

## 5. Conclusions

Our findings indicate that locally administrated antifungal drugs (micafungin and voriconazole) caused significant changes to the structure and frictional properties of the erythrocyte surface. There are some known adverse effects of micafungin treatment (e.g., microangiopathic hemolytic anemia, thrombocytopenia, microvascular thrombosis, and eryptosis), but no similar evidence exists regarding voriconazole. It should be highlighted that the present evidence of the possibility of eryptosis in peripheral blood was in our case examined according to a local drug-administration route. These effects occur in the long-term period after administration of the drugs, and represent a potential possibility of violation of tissue blood supply and the further development of negative side effects.

## Figures and Tables

**Table 1:** Roughness (R_q_) recorded for erythrocyte surfaces of three types of property maps.

Sample	Height	Deflection Error	Friction
Control (*n* = 20)	79.5(74.3;95.5) nm	12.9(10.5;15.5) mV	7.0(6.0;10.9) mV
ΜM (*n* = 38)	* 29.0(20.5;39.5) nm	** 7.0(5.0;9.6) mV	*** 11.0(8.3;15.5) mV
VM (*n* = 42)	* 26.5(22.0;40.25) nm	** 7.0(4.3;8.9) mV	**** 10.0(8.0;13.2) mV

Mann–Whitney U test: * p < 10^−6^, ** p < 0.001, *** p = 0.003301, **** p = 0.02668. The map size was 1 μm × 1 μm. The scanning step was 9.8 nm.

**Table 2:** Parameters of three Gauss peaks in the PSD for the deflection error map of the erythrocyte surfaces of different samples.

Sample	Peak 1	Peak 2	Peak 3	R^2^
Control	43 nm (59%)	82 nm (30%)	161 nm (11%)	0.996
ΜM	48 nm (59%)	94 nm (32%)	206 nm (8%)	0.995
VM	46 nm (64%)	91 nm (28%)	199 nm (8%)	0.990

## Data Availability

Not applicable.
